# Perceived social support and quality of life in endometrial cancer patients: a longitudinal study

**DOI:** 10.3389/fonc.2024.1447644

**Published:** 2024-08-02

**Authors:** Vincenzo Dario Mandato, Marcella Paterlini, Federica Torricelli, Elisa Rabitti, Valentina Mastrofilippo, Lorenzo Aguzzoli

**Affiliations:** ^1^ Obstetrics and Gynecological Oncology, Azienda Unità Sanitaria Locale (AUSL) - Istituto di Ricovero e Cura a Carattere Scientifico (IRCCS) di Reggio Emilia, Reggio Emilia, Italy; ^2^ Department of Obstetrics and Pediatrics, Azienda Unità Sanitaria Locale (AUSL) - Istituto di Ricovero e Cura a Carattere Scientifico (IRCCS) di Reggio Emilia, Reggio Emilia, Italy; ^3^ Laboratory of Translational Research, Azienda Unità Sanitaria Locale (AUSL) - Istituto di Ricovero e Cura a Carattere Scientifico (IRCCS) di Reggio Emilia, Reggio Emilia, Italy; ^4^ Psycho-oncology Unit, Azienda Unità Sanitaria Locale (AUSL) - Istituto di Ricovero e Cura a Carattere Scientifico (IRCCS) di Reggio Emilia, Reggio Emilia, Italy

**Keywords:** endometrial cancer, quality of life, social support, radiotherapy, physician-patient communication, well-being, laparoscopy, adjuvant therapy

## Abstract

**Objective:**

This study aimed to assess the influence of medical history, perceived physician-patient communication, and perceived social support on changes in the quality of life (QoL) during the first year of follow-up in patients undergoing surgery for endometrial cancer (EC), the most prevalent gynecological cancer in Western countries, especially in Central and Eastern Europe and North America.

**Methods:**

This prospective longitudinal study included 98 EC patients. All participants completed the Short Form 36 (SF-36) and the Multidimensional Scale of Perceived Social Support (MSPSS) one month and one year after surgery. Additionally, one month after surgery, they responded to a questionnaire designed by the researchers concerning the key aspects of physician-patient communication.

**Results:**

Our findings revealed that patients reporting high social support one month after surgery demonstrated significantly improved emotional well-being (EWB) at both one month and one year after the surgery, with statistically significant higher scores in the dimension of EWB (p<0.05). The support from a significant other at one year correlates with greater PF (p<0.005), fewer limitations due to physical health (p<0.05), less pain (p<0.05), less fatigue (p<0.05), and better general and EWB (p<0.05).

**Conclusion:**

This study underscores the significance of perceived social support for patients cross endometrial cancer. The multifaceted nature of social support, encompassing emotional assistance and information sharing, emerges as a pivotal factor aiding patients in confronting the challenges inherent to EC. This form of support contributes to bolstering psychological well-being and enhancing overall QoL.

## Introduction

Endometrial cancer (EC) is the most common gynecological cancer in Western countries particularly in Central and Eastern Europe and North America. In 2020, 417,000 new EC cases and 97,000 deaths were reported worldwide (1). In the last decade, its incidence has increased especially in women under the age of 50 (2). Moreover, the incidence of EC is rising in high-income countries, which may be attributable to high rates of obesity, physical inactivity, late menopause, and extended life expectancy. EC is usually diagnosed at early stage because it causes symptoms such as bleeding not related to menstruation and postmenopausal bleeding (3). Therefore, when diagnosed at early stage, EC usually has a favorable prognosis (77% 5-year overall survival [OS]), such that even fertility-sparing treatments are safe (4–7). Conversely, advanced or recurrent disease results in low response to chemotherapy and poor outcome (6–8). EC patients are potentially long-surviving patients, and therefore quality of life (QoL) is a highly relevant topic. In recent decades, QoL has become one of the main outcomes to ensure when choosing cancer treatment (9–11). Quality of Life is defined by the World Health Organization (WHO) as “an individual’s perception of their position in life in the context of the culture and value systems in which they live and in relation to their goals, expectations, standards and concerns” (12). QoL reflects the patient’s subjective assessment of all dimensions of their health experience, including physical health, psychological state, level of autonomy, social relationships, personal beliefs, and their relationships to important aspects of the environment (12). In patients suffering from diseases that could reduce survival, both the treatment and the disease itself can impair QoL (9). It has been well documented that many cancer patients report long-term psychological distress (13, 14). Standard treatments such as surgery, chemotherapy, and radiation therapy can impair QoL (9). Different surgical approaches can affect QoL differently. In particular, laparoscopy is associated with less pain, fewer complications and shorter hospital stay than laparotomy, with a better perception of physical well-being (15–17). Emotional well-being (EWB) in patients who have undergone surgery for EC is a multifaceted issue, profoundly influenced by social support (SS) and communication with healthcare providers. These variables are crucial in determining the patients’ overall QoL and their ability to cope with the aftermath of their diagnosis and treatment. Social support is a vital factor in the EWB of EC patients. It encompasses the emotional, informational, and practical assistance provided by family, friends, and healthcare professionals. Research by Pasek et al. (18) highlights that SS significantly impacts patients’ psychological health by reducing stress and enhancing their ability to manage illness-related challenges. Patients with robust social networks tend to report lower levels of anxiety and depression and exhibit better overall MH. A study by Chan et al. (19) further supports these findings, showing that family and friend support directly influences patients’ QoL. Effective SS leads to improved emotional resilience and a more positive outlook, which are crucial for recovery and long-term well-being. According to Smith-Bindman et al. (20), patients who feel understood and supported by their doctors are more likely to experience positive emotional states and lower levels of psychological distress. This underscores the importance of empathy and clear communication in medical practice, especially for patients dealing with life-threatening conditions like EC. Satisfactory information about cancer, the necessary treatment and long-term effects can reduce patients’ fears and anxieties, with a positive impact on QoL. Several studies have reported that cancer survivors who are satisfied with the information they received have a better health-related QoL as well as lower levels of depression and anxiety (21). Adequate information increases awareness in the decision-making process by decreasing stress factors and represents an important support factor in the diagnostic and therapeutic path of the disease (22). Similarly, the perception of having received good SS is also an important protective factor in mitigating the negative impact of stressful events and in developing greater resilience, favoring greater individual well-being and a better QoL (22, 23). In this study, we investigated the QoL of patients who underwent surgery for EC treatment. We investigated the change in QoL during the first year of follow-up based on perceived SS (PSS).

We hypothesized that medical history and PSS could have an effect on patients’ QoL and general well-being. The increase in the patients’ QoL during the study period could be mediated by the socio-personal characteristics of the patients themselves, their clinical history and the quality of PSS. We also wanted to verify whether a better perception of physician-patient communication is associated with a further increase in the well-being of the patients participating in the study.

## Methods

The aim of this prospective longitudinal study was to evaluate the impact of medical history, perceived physician-patient communication, and PSS on changes in QoL during the first year of follow-up of patients who had undergone surgery for EC. The standard treatment for EC includes total hysterectomy with bilateral salpingo-oophorectomy. The follow-up or adjuvant treatment is planned according to the risk factors of each case (6).

All EC patients treated at our hospital were evaluated for inclusion and exclusion criteria. Patients were eligible to participate if they had a histological diagnosis of EC, were age >18 years, if they had been treated surgically at our hospital, and if they were able to speak and understand the Italian language. Exclusion criteria were absence of histological diagnosis of EC, absence of standard surgical treatment (e.g. conservative treatment), and inability to speak and understand the Italian language. Self-administered QoL questionnaires were delivered to patients one month and one year postoperatively at the follow-up visit.

### Ethics approval and consent to participate

Study number 45/2012, date of approval by the Regional Ethics Committee 16/04/2012. Written informed consent was obtained from all individual participants included in the study.

### Data collection

The day of the gynecological check-up visit, a psychologist researcher formally contacted all patients who met the inclusion criteria to explain to them the study protocol and ask for their consent to participate in the study. Patients were included in the study only after obtaining a formal written informed consent. At enrollment, demographic and clinical data were recorded for all patients. At each check-up visit, participants received an envelope containing two questionnaires. They were asked to complete the first questionnaire one month after surgery and the second questionnaire one year after surgery. The investigators were required to provide potential participants with oral and written information about the aim and procedures of the study at enrollment.

### Measurement instruments

QoL was measured using the Short Form 36 (SF-36) (24) in the Italian version (25). The SF-36 comprises 36 items and provides scores for eight dimensions of physical and mental health (MH)-related QoL: physical functioning (PF), i.e., the extent to which health interferes with performance in everyday physical activities (e.g., carrying groceries, climbing stairs, and walking); physical role (PR) functioning, i.e., the degree to which health interferes with usual daily activities such as work, housework, or school; bodily pain (BP), i.e., the intensity of bodily pain and the extent to which it interferes with normal work; general health (GH), i.e., the current evaluation of personal health; vitality (VT), i.e., the degree to which a person feels full of energy or worn out and tired; social functioning (SF), i.e., the extent to which health had interfered with normal social activities like visiting friends during the past month; emotional role functioning (ER), i.e., the degree to which emotional problems impede every day activities such as work; and MH, i.e., the extent to which a person feels a positive or a negative mood. The scores range from 0 to 100, with higher scores at each dimension indicating better QoL. In the present study, Cronbach’s alpha values for the SF-36 dimensions ranged between 0.70 and 0.87.

Specifically, the present article focuses also on PSS that was measured using the Multidimensional Scale of Perceived Social Support (MSPSS), which was created by Zimet et al. (26) and includes 12 items (using a 5-point scale from ‘‘strongly disagree’’ to ‘‘strongly agree’’). The MSPSS is divided into three subscales: Friends, Family, and Significant Other, and therefore yields four scores: a total score (range 0–72) for the PSS and three scores for the different sources of PSS (range 0–24), namely, the patient’s friends, the patient’s family, and the patient’s significant other.

In addition, a new questionnaire specially created by the researchers for this study was administered. This questionnaire focused on the salient characteristics of physician-patient communication, designed to test contextual and interpersonal characteristics of the moment of communication itself (see **Supplementary Material**).

### Statistical analysis

All statistical analyses were performed using R software (version 4.2.1).

Differences in categorical and continuous variables were evaluated applying Fisher’s exact test and the ANOVA test, respectively. Differences were considered statistically significant with a p value <0.05.

## Results

The questionnaires were administered to 127 patients, but only 98 patients returned the questionnaires correctly completed one month and/or one year after surgery. Therefore, 98 patients were included in the analysis. The clinical characteristics of the patients included in this study are summarized in **Table 1**.

**Table 1:** Clinical characteristics of the patients.

	Overall (N=98)
Menopausal state	
Post-menopausal	64 (83.1%)
Pre-menopausal	13 (16.9%)
Misses	21
BMI	
Mean (SD)	28.6 (7.5)
Range	19.0 - 49.0
Misses	49
Comorbidies	
No	27 (42.2%)
Yes	37 (57.8%)
Misses	34
Histotype	
Endometrioid	82 (89.1%)
Hyperplasia	3 (3.3%)
Other	7 (7.6%)
Misses	6
Stadio. FIGO	
IA	74 (77.1%)
IB	20 (20.8%)
IIIC	2 (2.1%)
Misses	2
Grading	
G1	55 (61.1%)
G2	28 (31.1%)
G3	7 (7.8%)
Misses	8
Surgery	
Colposcopy	1 (1.0%)
Laparoscopy	62 (63.9%)
Laparotomy	32 (33.0%)
Laparoscopy+Laparotomy	2 (2.1%)
Misses	1
Pelvic lymphadenectomy	
No	65 (66.3%)
Yes	33 (33.7%)
Lomboaortic lymphadenectomy	
No	94 (95.9%)
Yes	4 (4.1%)
Presence of positive lymph nodes	
No	95 (97.9%)
Yes	2 (2.1%)
Misses	1
Surgical time (minutes)	
Mean (SD)	132.8 (45.1)
Misses	3
Days of hospitalization	
Mean (SD)	5.3 (2.0)
Misses	4
Postoperative complications	
No	81 (86.2%)
Yes	13 (13.8%)
Misses	4
Extra visits	
No	87 (91.6%)
Yes	8 (8.4%)
Misses	3

SD, standard deviation.

The results of the scores obtained on the SF-36 and MSPSS questionnaires showed that patients who claim to have high social support at one month have more accentuated EWB both one month and one year after the surgery (**Table 2**), with statistically significant higher scores in the dimension of EWB (p<0.05). Patients who reported better SS one year after surgery also scored high on the GH dimension of the SF-36 (p<0.05) (**Table 2**). Particularly, those who reported having high family support had a higher SF score at one month after the surgery (p<0.05) (**Table 3**). Furthermore, one year after the surgery, these patients reported fewer limitations due to emotional problems than those who believe they have had medium to low family support (**Table 3**). Patients who reported having high family support at one year showed fewer fatigue problems and better SF (**Table 3**).

**Table 2:** SF36 scores comparison in patients who reported a different general social support.

		Social support Total_scale 1 Month			Social support Total_scale 1 Year		
		Low-medium (N=23)	High (N=74)	p value	Low-medium (N=22)	High (N=72)	p value
**SF36** **1Month**	**Physical functioning**			0.104			
	Mean (SD)	73.6 (20.5)	64.2 (24.4)				
	**Role limitation due to physical health**			0.822			
	Mean (SD)	20.5 (37.5)	18.6 (33.1)				
	**Role limitation due to emotional problems**			0.368			
	Mean (SD)	36.4 (42.3)	45.6 (42.2)				
	**Energy/Fatigue**			0.529			
	Mean (SD)	49.0 (18.0)	52.0 (18.6)				
	**Emotional well-being**			**0.012**			
	Mean (SD)	50.0 (18.7)	62.0 (18.6)				
	**Social functioning**			0.106			
	Mean (SD)	61.5 (29.1)	71.7 (24.5)				
	**Pain**			0.770			
	Mean (SD)	58.5 (22.2)	60.4 (26.9)				
	**General health**			0.334			
	Mean (SD)	56.4 (25.4)	61.3 (18.6)				
**SF36** **1 Year**	**Physical functioning**			0.478			0.085
	Mean (SD)	73.9 (27.8)	78.3 (24.4)		68.3 (30.6)	79.1 (23.9)	
	**Role limitation due to physical health**			0.645			0.267
	Mean (SD)	62.0 (43.2)	66.7 (42.4)		55.7 (41.5)	67.4 (43.3)	
	**Role limitation due to emotional problems**			0.956			0.910
	Mean (SD)	68.1 (44.4)	67.6 (41.2)		69.7 (43.6)	68.5 (41.1)	
	**Energy/Fatigue**			0.077			0.264
	Mean (SD)	51.7 (21.3)	60.1 (18.8)		53.6 (18.5)	59.0 (20.0)	
	**Emotional well-being**			**< 0.001**			0.134
	Mean (SD)	52.9 (23.5)	69.4(15.6)		59.8 (18.8)	66.8 (19.0)	
	**Social functioning**			0.473			0.103
	Mean (SD)	78.3 (24.1)	82.2 (21.5)		75.1 (24.0)	84.0 (21.4)	
	**Pain**			0.182			0.120
	Mean (SD)	68.4 (27.8)	76.5 (24.0)		66.3 (27.0)	76.1 (25.1)	
	**General health**			0.363			0.010
	Mean (SD)	59.0 (22.1)	63.6 (20.9)		52.3 (19.7)	65.6 (20.9)	

Scores collected from both questionnaires at 1 month and 1 year from surgery were evaluated and integrated into statistical analyses. SD, standard deviation.

Bold and colored text means significant p-value.

**Table 3:** SF36 scores comparison in patients who reported a different family support.

		Social support Family subscale 1 Month			Social support Family subscale 1 Year		
		Low-Medium (N=23)	High (N=74)	p value	Low- Medium (N=22)	High (N=72)	p value
**SF36** **1 Month**	**Physical functioning**			0.676			
	Mean (SD)	63.8 (28.1)	66.9 (23.4)				
	**Role limitation due to physical health**			0.618			
	Mean (SD)	14.6 (31.0)	19.9 (34.7)				
	**Role limitation due to emotional problems**			0.257			
	Mean (SD)	30.6 (41.4)	45.5 (42.4)				
	**Energy/Fatigue**			0.194			
	Mean (SD)	44.5 (10.4)	52.3 (19.2)				
	**Emotional well-being**			0.183			
	Mean (SD)	52.4 (19.9)	60.6 (19.0)				
	**Social functioning**			**0.006**			
	Mean (SD)	50.2 (22.5)	72.1 (25.3)				
	**Pain**			0.381			
	Mean (SD)	54.3 (22.9)	61.2 (26.0)				
	**General health**			0.170			
	Mean (SD)	52.9 (21.7)	61.6 (20.0)				
**SF36** **1 Year**	**Physical functioning**			0.833			0.153
	Mean (SD)	75.8 (32.5)	77.5 (24.2)		68.7 (34.5)	78.5 (23.2)	
	**Role limitation due to physical health**			0.615			0.402
	Mean (SD)	59.6 (45.1)	66.0 (42.3)		56.9 (42.7)	66.4 (43.1)	
	**Role limitation due to emotional problems**			**0.046**			0.384
	Mean (SD)	46.2 (48.2)	71.1 (40.2)		61.1 (44.7)	70.6 (40.7)	
	**Energy/Fatigue**			0.108			**0.019**
	Mean (SD)	50.0 (19.7)	59.5 (19.5)		48.12(22.6)	60.1 (18.3)	
	**Emotional well-being**			0.117			0.060
	Mean (SD)	57.5 (20.2)	66.6 (18.8)		57.6 (18.6)	67.0 (18.9)	
	**Social functioning**			0.122			**0.008**
	Mean (SD)	72.2 (24.6)	82.5 (21.5)		69.6 (28.5)	84.8 (19.6)	
	**Pain**			0.690			0.222
	Mean (SD)	76.8 (22.4)	73.8 (25.6)		67.1 (29.6)	75.4 (24.7)	
	**General health**			0.619			0.077
	Mean (SD)	59.7 (25.8)	62.9 (20.6)		54.4 (22.0)	64.3 (20.8)	

Scores collected from both questionnaires at 1 month and 1 year from surgery were evaluated and integrated into statistical analyses. SD, standard deviation.

Bold and colored text means significant p-value.

**Figure 1:**
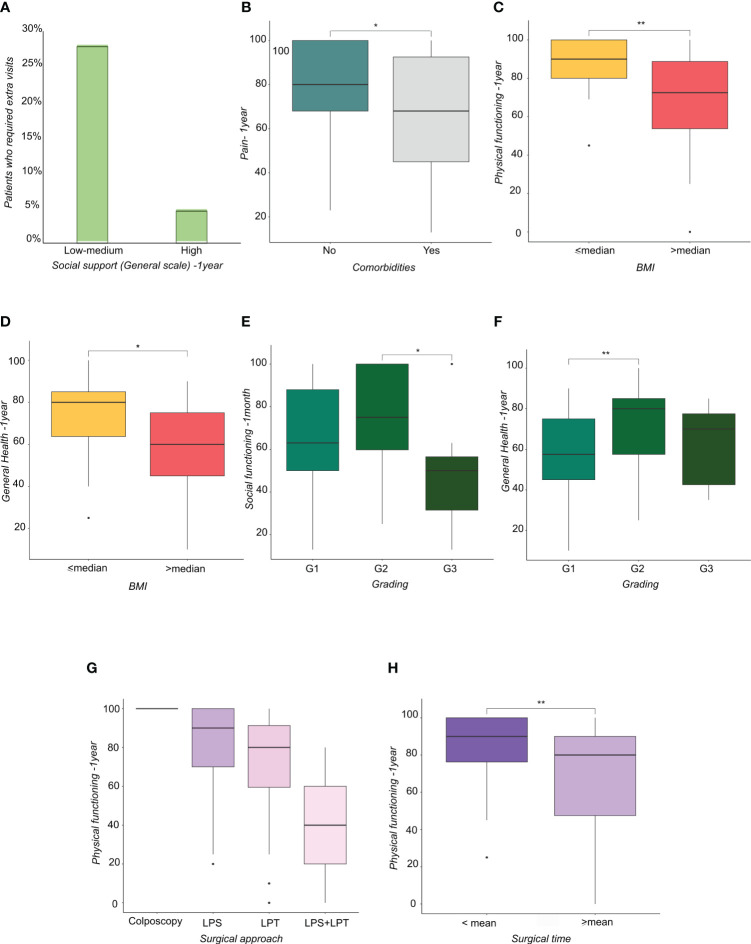
**(A)** Histograms representing the percentage of patients who required extra visits subdivided by social support perception. **(B)** Boxplots representing the distribution of Pain score at one year from surgery in patients subdivided by the presence of comorbidities. **(C, D)** Boxplots representing the distribution of **(C)** physical functioning score at one year from surgery and **(D)** general health at one year from surgery in patients subdivided by Body Mass Index-BMI (lower or higher than median value). **(E, F)** Boxplots representing the distribution of **(E)** Social functioning score at one month and **(F)** General Health score at one year from surgery in patients subdivided by tumor grading. **(G, H)** Boxplots representing the distribution of physical functioning score at one year from surgery in patients subdivided by **(G)** surgical approach and **(H)** surgical time (lower or higher than mean time). * <0.05, **<0.01.

The scores also showed that support from a significant other at one year correlates with greater PF (p<0.005), fewer limitations due to physical health (p<0.05), less pain (p<0.05), less fatigue (p<0.05) and better general and EWB (p<0.05) (**Table 4**). Moreover, patients who had high friend support at one month reported a higher well-being score than those who felt unsupported by others (p<0.05). Patients who felt they had a high degree of friend support at one month reported a higher well-being score than those who felt unsupported by others (p<0.05) (**Table 5**). Social support in its various forms (friend support, significant other support, etc.) correlated with a lower request for extra medical visits in the first year following the surgery (**Figure 1A**). Correlating clinical data to SF-36 and MSPSS results showed that patients with comorbidities (hypertension, diabetes, hypercholesterolemia) reported higher overall pain, corresponding to a lower score, one year after surgery (**Figure 1B**). Regarding the body max index (BMI) effect, patients with BMI higher than the median reported a significantly lower score of physical functioning (**Figure 1C**) and GH (**Figure 1D**) at one year from surgery.

**Table 4:** SF36 scores comparison in patients who reported a different support from another significant person.

		Social support Other subscale 1 Month			Social support Other subscale 1 Year		
		Low-Medium (N=18)	High (N=79)	p value	Low- Medium (N=13)	High (N=81)	p value
**SF36** **1 Month**	**Physical functioning**			0.560			
	Mean (SD)	69.4 (25.6)	65.7(23.5)				
	**Role limitation due to physical health**			0.568			
	Mean (SD)	14.7 (26.6)	19.9 (35.4)				
	**Role limitation due to emotional problems**			0.273			
	Mean (SD)	33.3 (42.5)	45.7 (42.0)				
	**Energy/Fatigue**			0.495			
	Mean (SD)	48.4 (21.7)	51.9 (17.7)				
	**Emotional well-being**			**0.045**			
	Mean (SD)	50.8 (23.2)	61.2 (17.8)				
	**Social functioning**			0.068			
	Mean (SD)	59.0 (30.2)	71.6 (24.4)				
	**Pain**			0.946			
	Mean (SD)	60.4 (26.4)	59.9 (25.8)				
	**General health**			0.089			
	Mean (SD)	52.6 (28.1)	61.9 (17.9)				
**SF36** **1 Year**	**Physical functioning**			0.444			**< 0.001**
	Mean (SD)	73.1 (25.2)	78.2 (25.2)		51.7 (31.1)	80.6 (22.7)	
	**Role limitation due to physical health**			0.626			**0.043**
	Mean (SD)	61.1 (43.1)	66.6 (42.5)		42.3 (46.1)	68.2 (41.6)	
	**Role limitation due to emotional problems**			0.748			0.100
	Mean (SD)	64.8 (45.0)	68.4 (41.3)		51.2 (48.4)	71.6 (39.9)	
	**Energy/Fatigue**			0.430			**0.017**
	Mean (SD)	54.7 (18.5)	58.8 (20.0)		45.8 (21.4)	59.7 (18.8)	
	**Emotional well-being**			0.054			**0.046**
	Mean (SD)	57.6 (19.3)	67.2 (18.7)		55.4 (20.2)	66.8 (18.6)	
	**Social functioning**			0.314			0.311
	Mean (SD)	76.5 (24.9)	82.3 (21.4)		76.1 (25.7)	82.8 (21.7)	
	**Pain**			0.630			**0.006**
	Mean (SD)	71.9 (27.9)	75.1 (24.5)		55.7 (26.5)	76.7 (24.6)	
	**General health**			0.366			**0.033**
	Mean (SD)	58.4 (25.2)	63.4 (20.1)		50.8 (21.5)	64.3 (20.8)	

Scores collected from both questionnaires at 1 month and 1 year from surgery were evaluated and integrated into statistical analyses. SD, standard deviation.

Bold and colored text means significant p-value.

**Table 5:** SF36 scores comparison in patients who reported a different support from friends.

		Social support Friends subscale 1 Month			Social support Friends subscale 1 Year		
		Low-Medium (N=18)	High (N=79)	p value	Low-Medium (N=13)	High (N=81)	p value
**SF36** **1** **MONTH**	**Physical functioning**			0.841			
	Mean (SD)	66.9 (22.7)	65.9 (24.8)				
	**Role limitation due to physical health**			0.504			
	Mean (SD)	16.3 (32.8)	21.0 (35.0)				
	**Role limitation due to emotional problems**			0.718			
	Mean (SD)	41.7 (42.6)	44.8 (42.2)				
	**Energy/Fatigue**			0.077			
	Mean (SD)	47.2 (16.1)	54.1 (19.4)				
	**Emotional well-being**			**0.047**			
	Mean (SD)	54.6 (18.1)	62.6 (19.3)				
	**Social functioning**			0.374			
	Mean (SD)	66.5 (26.7)	71.3 (25.2)				
	**Pain**			0.106			
	Mean (SD)	54.9 (25.0)	63.6 (26.0)				
	**General health**			0.152			
	Mean (SD)	56.6 (22.9)	62.7 (18.0)				
**SF36** **1** **YEAR**	**Physical functioning**			0.531			0.237
	Mean (SD)	75.4 (26.5)	78.7 (24.2)		73.7 (27.37)	80.17 (24.37)	
	**Role limitation due to physical health**			0.765			0.818
	Mean (SD)	64.0 (43.0)	66.7 (42.3)		66.9 (40.7)	64.8 (44.8)	
	**Role limitation due to emotional problems**			0.269			0.223
	Mean (SD)	73.2 (40.3)	63.6 (42.7)		75.2 (37.9)	64.6 (43.8)	
	**Energy/Fatigue**			0.336			0.294
	Mean (SD)	55.8 (20.9)	59.7 (18.7)		55.814 (19.668)	60.102 (19.242)	
	**Emotional well-being**			0.114			0.396
	Mean (SD)	61.7 (23.0)	68.0 (15.2)		63.6 (22.4)	67.0 (15.5)	
	**Social functioning**			0.709			0.975
	Mean (SD)	80.3 (24.0)	82.0 (20.8)		82.7 (22.1)	82.8 (21.3)	
	**Pain**			0.540			0.187
	Mean (SD)	72.7 (25.3)	75.9 (25.0)		69.8 (25.6)	77.0 (26.1)	
	**General health**			0.255			0.092
	Mean (SD)	59.5 (23.6)	64.6 (19.1)		58.4 (22.3)	65.9 (19.9)	

Scores collected from both questionnaires at 1 month and 1 year from surgery were evaluated and integrated into statistical analyses. SD, standard deviation.

Bold and colored text means significant p-value.

**Figure 2:**
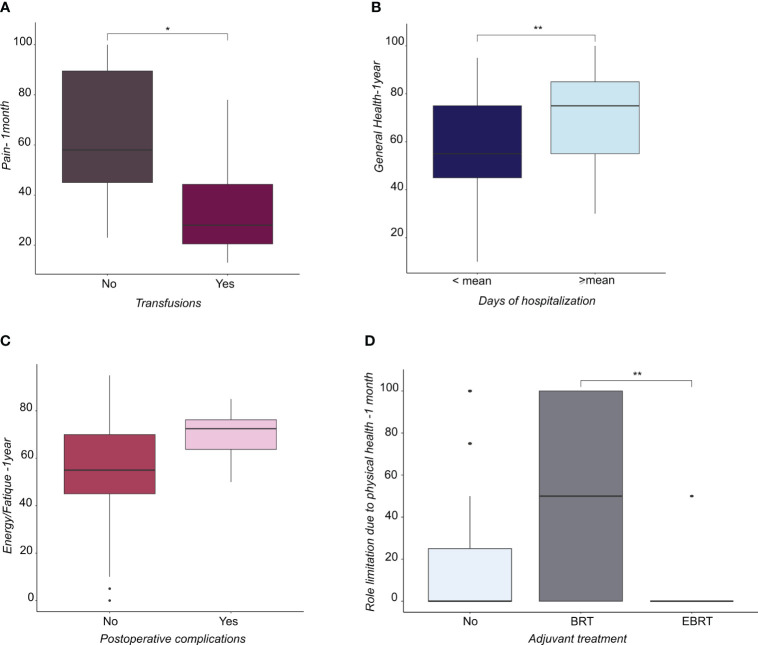
**(A)** Boxplots representing the distribution of pain score at 1 month from surgery in patients subjected or not to transfusions. **(B)** Boxplots representing the distribution of General Health score at 1 year from surgery in patients subdivided by days of hospitalization (lower or higher than mean). **(C)** Boxplots representing the distribution of Energy/fatique score at 1 year from surgery in patients that underwent or not postoperative complication. **(D)** Boxplots representing the distribution of the score related to the role limitation due to physical health at 1 month from surgery in patient not subjected to adjuvant treatment or subjected to brachytherapy (BRT) or external beam radiation therapy (EBRT). * <0.05, **<0.01.

Patients with grade (G) 3 EC reported a lower score in SF compared with patients with G2 EC (**Figure 1E**), whereas patients with G2 EC reported a higher score in GH compared with patients with G1 EC (**Figure 1F**). Regarding surgical treatment, patients who received laparoscopy converted to laparotomy (**Figure 1G**) and patients who had a longer operation time (**Figure 1H**) reported a lower PF score at one year. Patients with a longer hospital stay reported a higher GH score at one year after treatment, showing that they felt better than patients who had a shorter hospital stay (**Figure 2A**). Similarly, patients with postoperative complications reported a higher Energy/Fatigue score one year after treatment, showing that they felt stronger than patients without complications (**Figure 2B**). The results also showed that patients who received brachytherapy (BRT) had a significantly lower role limitation in regard to PF (corresponding to a higher score) one month from surgery than patients who underwent both BRT and external beam radiation therapy (EBRT) (p<0.05) (**Figure 2C**). Regarding physician-patient communication, 59.8% of the patients reported that they were accompanied by a family member at the time the diagnosis was communicated. In 75.3% of cases, the communication of the diagnosis took place in the gynecological oncology clinic and the patients responded that the time devoted to communication was sufficient (85.6%). It was also shown that most of the participants (82.5%) found an empathetic and supportive attitude, and 93.8% felt supported in their care. 84.5% of participants said that the physicians communicated in clear language, and 91.8% of patients reported that they felt their privacy was protected (91.8%).

## Discussion

The findings of this study reveal a noteworthy trend, namely that patients who perceived robust SS, both at the one-month and one-year junctures following the surgery, showed higher levels of EWB. In particular, high SS at one month is associated with a more accentuated EWB at both one month and one year after the surgery, with statistically significant scores in the dimension of EWB (p<0.05). Thus, the positive effect of high SS in the immediate postoperative period was maintained even after one year. Moreover, high PSS one year after surgery also was associated with a high score on the GH dimension of the SF-36 (p<0.05). The lack of social contacts, institutional connections and community involvement constitutes social isolation (27, 28). Social isolation has been associated with tumor metastasis, chemoresistance, resistance to radiotherapy and suppression of immune defense (29). Social isolation of cancer patients is associated with reduced OS therefore SS should be guaranteed and promoted in all cancer patients even in EC patients, generally characterized by a better prognosis. EC patients are typically obese, and high BMI and smoking have been reported to affect EWB (30, 31). In our study, the effect of BMI on EWB was confirmed, and in particular, patients with BMI higher than the median reported significantly lower scores of PF and GH at one year from surgery. In a recent study, Karataşli et al. reported that high BMI patients had lower PF scores (32). In a previous systematic review, high BMI showed detrimental effects on physical, social, and role functioning but not on emotional or cognitive functioning (33). Although there is still debate as to whether BMI can influence the risk of EC recurrence (34, 35), providing indications for making lifestyle and dietary changes could be useful for improving QoL regardless of the effect on oncological outcomes (30).

Socioeconomic status (SES) and adjuvant therapy can also affect the EWB of EC patients (36, 37). Patients with a low and intermediate SES reported improved emotional functioning over time, while patients with a high SES reported a higher but stable emotional functioning (36). A recent study reported that BRT is associated with higher EWB than EBRT, and with no lasting effects on emotional and functional health. In contrast, EC patients were still physically affected for years after completion of EBRT (37). In our study, patients who received EBRT combined with BRT showed worse physical role functioning than patients who received only BRT one month after treatment. In more aggressive gynecological cancers such as ovarian cancer, also multiple recurrences significantly decrease mean EWB; on the contrary PWB and FWB were above population norms because of the high levels of social well-being, with over 85% of each group reporting substantial emotional support from their families (38). Patients and their families can be particularly vulnerable when diagnosed with cancer. Cancer patients often turn to their family members to manage their psychological well‐being. The support of family members for their EC patient is essential to guarantee any home care but also to facilitate the resumption of routine daily activities. Unfortunately, cancer patients often have difficulty talking about the illness to their family members, and family members may, in the long run, suffer from psychological distress as a result of their cancer patient’s illness. Therefore, healthcare professionals must communicate effectively with patients and their families and teach patients and families to communicate with each other even in the event of bad news. Furthermore, it is necessary to support the psychological well-being of people accompanying cancer patients because the psychological well-being of cancer patients is closely connected with that of the people accompanying them (39, 40). In our study, those who reported having high family support had a higher SF score at one month after the surgery (p<0.05) (**Table 3**). Furthermore, one year after the surgery, there were fewer limitations due to emotional problems than those who believed they had had medium to low family support (**Table 3**). Other studies have shown that lacking emotional support from expected sources such as family and friends resulted in loneliness and anger (38). A comprehensive analysis of the SF-36 and MSPSS outcomes underscores a compelling association between high SS, notably familial, and a spectrum of favorable outcomes encompassing improved SF, diminished pain perception, reduced fatigue, and enhanced overall general and EWB. Intriguingly, a stronger perception of SS also coincides with a reduction in the need for supplementary medical visits during the year after the surgery. EC patients represent a large portion of patients diagnosed with gynecological cancers, with a large investment of resources. Several studies have shown that minimal in-person or telephone follow-ups can be effective in ensuring adequate surveillance, eliminating unnecessary care (41, 42). Promoting adequate support from the beginning of treatment could be further useful in optimizing resources allocated to oncological follow-ups and enabling healthcare cost savings. Examining the intricate interplay between clinical data and QoL, our study illuminates distinct correlations. Patients grappling with comorbidities report a heightened prevalence of general pain one year after surgery, highlighting the complex interaction between underlying medical conditions and sustained discomfort. Conversely, individuals contending with postoperative complications paradoxically exhibit elevated scores in the Energy/Fatigue dimension, indicative of an augmented sense of vitality. It is conceivable that overcoming a particularly critical condition such as postoperative complications requiring a longer hospital stay can foster, once overcome, greater self-confidence and greater vitality. However, this vitality is juxtaposed by lower scores in the PF dimension, emphasizing the nuanced trade-offs inherent in the relationship between clinical data and patient QoL. Regarding surgical treatment, patients who received laparoscopy converted to laparotomy and patients who had a longer operation time reported a lower PF rate at one year. According to the literature, EC patients undergoing laparoscopy converted to laparotomy have longer operating times, more blood loss, and more intraoperative and postoperative complications (43–45).

## Conclusions

According to previous literature (9, 11, 46), this study underscores the significance of PSS for EC patients. The multifaceted nature of SS, encompassing emotional assistance and information sharing, emerges as a pivotal factor aiding patients in confronting the challenges inherent to EC. This form of support contributes to bolstering psychological well-being and enhancing overall QoL.

Ensuring the holistic well-being of EC patients necessitates the provision of comprehensive medical, psychological, and SS throughout the trajectory of the illness.

A significant aspect illuminated by this study pertains to the profound impact of the communication modality on the psychological well-being of women diagnosed with EC. Notably, those who receive a diagnosis accompanied by empathetic communication, comprehensive tumor explanations, and elucidation of available treatment options tend to feel more supported during their treatment journey. Moreover, at the time of planning the surgery, counseling should be provided to improve lifestyle and eating habits.

Sharing treatment strategies with the relative risks and benefits allows for the creation of a therapeutic alliance, obtaining the maximum commitment from patients even in the face of the unforeseen events that each therapeutic path may present. This approach contributes to alleviating anxiety and stress associated with the diagnosis and the perceived loss of control over one’s life. On the contrary, a lack of informational support can result in significant uncertainty and fear (41).

The findings of our study not only emphasize the positive influence of social support from loved ones on the psychological well-being of women with EC, but also underscore the pivotal role of healthcare professionals’ communication in shaping the perception of the disease experience. Women who feel supported and understood experience reduced isolation and an enhanced ability to confront the challenges linked to their illness. This aspect warrants further investigation.

Assessing and addressing issues related to QoL constitutes an integral facet of modern medical care. Providing compassionate care to the patient, in conjunction with addressing the cancer itself, necessitates an evolving approach aimed at preserving and enhancing both the quality and quantity of life. Social support should be considered an essential component of health care, as it helps improve patients’ resilience and QoL during their cancer journey (36).

The evaluation of SS sources should be an integral part of treatment planning, particularly within the family context. Hence, involving spouses and other family members in relevant courses or strategies is recommended. Additionally, healthcare professionals play a pivotal role as sources of SS for women with cancer, necessitating a heightened understanding of these women’s unique experiences.

Although the communicative aspect was explored via a non-validated multiple-choice questionnaire, descriptive analysis suggests that a favorable perception of physician-patient communication (empathy, time allocation, setting, presence of a significant other) can positively impact the patient’s illness experience. These descriptive results underscore the importance for healthcare personnel to prioritize communicative and relational aspects when communicating the diagnosis and to inquire whether the patient desires information in the presence of a designated caregiver.

### Strengths, weaknesses, and future prospects

A key strength of this study lies in its emphasis on SS and the value attributed to patient-healthcare provider communication within the medical journey and illness experience of women with EC. Another notable strength is the discrete analysis of distinct sources of SS, facilitated by the unique instrument chosen for this study. Unfortunately, we do not have data for all patients, so the lack may have weakened our analysis. Any comorbidities or medications may have influenced our variables. However, a principal limitation of the research pertains to the modest sample size and the absence of correlation between responses to the communication questionnaire and data on QoL. Future studies could address these limitations by incorporating larger sample sizes and focusing on the influence of perceived physician-patient communication on psycho-EWB. Although most studies tested patients at four and six months, we decided to administer the questionnaires one year later, to avoid possible effects due to adjuvant therapies following surgery. We hypothesized that one year was a long enough period to evaluate the achievement of emotional balance.

In conclusion, this study provides valuable insights into the pivotal roles of EWB of EC patients is intricately linked to the level of SS they receive and the quality of their communication with healthcare providers. Strengthening these areas can lead to substantial improvements in their MH and overall QoL. The quality of care should always guarantee psychological well-being. Our study highlights that the quality of care already depends on the way in which the diagnosis and oncological pathways are communicated.

In a period in which public health resources are always scarce, investing in tools such as effective communication useful for improving assistance and at the same time reducing health care costs becomes essential (47). These findings underscore the need for comprehensive, patient-centered care that acknowledges the multidimensional facets of patient well-being and the nuanced impact of interpersonal interactions.

## Data availability statement

The original contributions presented in the study are included in the article/**Supplementary Material**, further inquiries can be directed to the corresponding author/s.

## Ethics statement

The studies involving humans were approved byArea Vasta Emilia Nord- Reggio Emilia. The studies were conducted in accordance with the local legislation and institutional requirements. The participants provided their written informed consent to participate in this study.

## Author contributions

ViM: Conceptualization, Investigation, Methodology, Writing – original draft, Writing – review & editing. MP: Conceptualization, Investigation, Methodology, Writing – original draft, Writing – review & editing. FT: Data curation, Formal Analysis, Writing – original draft. ER: Writing – original draft, Writing – review & editing. VaM: Data curation, Investigation, Writing – original draft, Writing – review & editing. LA: Investigation, Supervision, Writing – review & editing.
