# Supplementary material for: Perceived social support and quality of life in endometrial cancer patients: a longitudinal study
Source: Front Oncol. 2024 Aug 2;14:1447644. doi: 10.3389/fonc.2024.1447644 (PMC11327120; doi:10.3389/fonc.2024.1447644)
Supplement: Supplementary file 1 [file DataSheet_1.docx]

**QUESTIONNAIRE ON THE PERCEPTION OF EXPERIENCE RELATING TO THE COMMUNICATION OF GYNECOLOGICAL ONCOLOGICAL DIAGNOSIS:**

1. **Who was present when the diagnosis was communicated?**
2. **Relative**
3. **Friend**
4. **Other…**
5. **Nobody**
6. **Who was the diagnosis communicated by?**
7. **Surgeon**
8. **Hospital gynecologist**
9. **Nurse**
10. **Other…**
11. **Who did you receive the information about your disease from?**
12. **Department doctor**
13. **Nurse**
14. **General practitioner**
15. **Other…**
16. **Where was the diagnosis communicated?**
17. **Ward corridor**
18. **Medical clinic**
19. **Hospital room**
20. **Doctor’s office**
21. **Other…**
22. **Did you feel your privacy was protected?**
23. **Yes**
24. **No**
25. **Enough**
26. **Other…**
27. **Have you been given sufficient time to ask questions about your desease/diagnosis?**
28. **Yes**
29. **No**
30. **Enough**
31. **Other…**
32. **Did the information you received seem sufficient to you?**
33. **Yes**
34. **No**
35. **Enough**
36. **Other…**
37. **Was the language used to communicate the diagnosis clear and understandable?**
38. **Yes**
39. **No**
40. **Enough**
41. **Other…**
42. **The time elapsed between communication of the diagnosis and surgery was:**
43. **Excessive**
44. **Correct**
45. **I wish more time had passed**
46. **I wish less time had passed**
47. **Other…**
48. **Would you have preferred to have more meetings to discuss diagnosis and treatment before surgery?**
49. **Yes**
50. **No**
51. **Other…**
52. **Do you think that the attitude among the operators you met was coherent and homogeneous?**
53. **Yes**
54. **No**
55. **In part**
56. **Other…**
57. **Does what the doctor explained to you about how you would feel after the surgery correspond to how you actually felt?**
58. **Yes**
59. **No**
60. **In part**
61. **Other…**
62. **How did you perceive the attitude of the person who communicated the diagnosis to you?**
63. **Empathic/comfortable**
64. **Cold/distant**
65. **Other…**
66. **During the journey you faced, did you feel that the operators (doctors, nurses, head nurse, etc etc...) were taking "care" of you, or was it a cold administration of information and treatment?**
67. **I felt welcomed**
68. **Professional interest only**
69. **Other…**
70. **Is there anything you weren't asked in the questionnaire that you would like to add?**

**Write if you want your Personal Observations**

**…………………………………………………………………………………………………………………………………………………………………………………………………………………………………………………………………………………………………………………………………………………………………………**
